# Postural control during sit-to-stand movement and its relationship with upright position in children with hemiplegic spastic cerebral palsy and in typically developing children

**DOI:** 10.1590/bjpt-rbf.2014.0069

**Published:** 2015

**Authors:** Silvia L. Pavão, Adriana N. Santos, Ana B. Oliveira, Nelci A. C. F. Rocha

**Affiliations:** 1 Departamento de Fisioterapia, Universidade Federal de São Carlos (UFSCar), São Carlos, SP, Brazil

**Keywords:** hemiplegic spastic cerebral palsy, postural control, sit-to-stand movement, children, functional activity, rehabilitation

## Abstract

**OBJECTIVE:**

The purpose of this study was to compare postural control in typically developing (TD) children and children with cerebral palsy (CP) during the sit-to-stand (STS) movement and to assess the relationship between static (during static standing position) and dynamic postural control (during STS movement) in both groups.

**METHOD:**

The center of pressure (CoP) behavior of 23 TD children and 6 children with spastic hemiplegic CP (Gross Motor Function Classification System [GMFCS] I and II) was assessed during STS movement performance and during static standing conditions with the use of a force plate. The data obtained from the force plate were used to calculate CoP variables: anteroposterior (AP) and mediolateral (ML) amplitudes of CoP displacement and the area and velocity of CoP oscillation.

**RESULTS:**

According to the *Mann-Whitney* test, children with CP exhibited higher CoP values in all of the analyzed variables during the beginning of STS movement. Pearson's correlation verified a positive correlation between the CoP variables during both static conditions and the performance of STS movement.

**CONCLUSIONS:**

Children with spastic hemiplegic CP present major postural oscillations during the beginning of STS movement compared with typical children. Moreover, the observed relationship between postural control in static and dynamic conditions reveals the importance of body control in the static position for the performance of functional activities that put the body in motion, such as STS movement.

## Introduction

Children with cerebral palsy (CP) exhibit neuromotor disorders^1^^-^ 3, among which postural control deficits have a central role^4^^,^ 5. The postural control deficits observed in this population result in important limitations to daily functional activities^6^ because postural alignment and stability are requirements for voluntary movement^7^^,^ 8.

Although there is a wide description of postural control in CP in the literature^4^^,^ 9, most studies assessed children in static posture^10^^-^ 12 and used samples formed mainly of children with spastic diplegia CP^9^^,^ 11 ^,^ 12. These studies assessed the importance of postural control disorders, such as higher values of postural oscillation in CP, modifications of the muscle recruitment order to maintain stability, and higher rates of agonist-antagonist muscle coactivation. Few studies have assessed the postural control of children with spastic hemiplegia CP, especially during functional activities^13^. This lack of studies is more evident when assessing postural control during a change in posture, such as sit-to-stand movement (STS). Limitations of STS movement seriously affect an individual's daily functional activities.

STS movement is performed numerous times in the daily routine, demanding a stable coordination between the body segments to control the transition of the body from sitting to a standing posture. Furthermore, it is an antigravity movement involving the transition from a more stable position (seated) to a less stable one (standing)^14^. Therefore, it is a challenging movement with great biomechanical demands, requiring high levels of knee and ankle extension movements^15^.

For a better understanding, STS movement is divided into phases. The division into three phases is the most commonly used, i.e., the preparation phase, including the beginning of anterior trunk flexion to maximum flexion, when the body starts the seat-off; the rising phase, from the seat-off (maximum anterior flexion of the trunk) to standing posture; and the stabilization phase, which involves maintaining the body in a quasi-stationary position^16^^,^ 17.

The authors observed that children with CP have altered ability to initiate the lower limb joint movements necessary to assume the standing posture^18^. These children also have higher variability in body alignment strategies during STS movement, using greater trunk flexion in an attempt to bring their center of pressure (CoP) closer to the support base to gain stability, which could be a consequence of a postural control impairment^18^^-^ 20.

Studying postural control in individuals with postural impairments caused by voluntary movement^5^ allows for conditions to be reproduced that are closer to those experienced by the children in their daily routine and, thus, better understanding the postural control impairments associated with CP. Therefore, this study aimed to compare the postural control of typically developing children with that of children with spastic hemiplegia CP during each one of the three phases of STS movement. In addition, this study assessed the relationship between static (during standing posture) and dynamic postural control (during STS movement) in the assessed groups.

Children with CP exhibit increased postural oscillation when performing STS movement, reflecting difficulty in assuming the standing position. In addition, taking into account the importance of postural control while in the standing position to perform motor tasks and to perform functional activities in the daily routine^8^^,^ 12, one expects the postural control in the static standing condition to be directly related to the postural control during STS movement in both of the assessed groups. These expectations emphasize the importance of static control in the performance of daily functional activities, such as the STS movement.

## Method

Participants

Children were recruited from rehabilitation and childcare-specialized centers. The children were included in the study after their guardians signed the Informed Consent form. This study was approved by the Ethics Committee on Human Research of the *Universidade Federal de São Carlos* (UFSCar), São Carlos, SP, Brazil (Opinion No 363/2010).

Two groups were assessed. The control group consisted of 23 typically developing children between the ages of 5 to 12 years (mean=8.3±2.15). Patients with orthopedic disorders of the lower limbs or neurological, cardiovascular or systemic disorders that could limit the participants' level of physical activity were excluded from the study.

The experimental group consisted of 6 children with CP, all with spastic hemiplegia, Gross Motor Function Classification System (GMFCS) level I and aged between 5 and 12 years (mean=8.2±2.5). The inclusion criteria for the experimental group were: (a) the ability to follow simple verbal commands, assessed by the child's ability to follow the instructions to get up from a chair when asked; (b) the ability to independently assume the standing position; (c) 0 (no increase in muscle tone) or 1 score (slight increase in muscle tone) on a muscle tone classification, according to the modified Ashworth scale; (d) participation in a twice a week physical therapy program for at least 6 months. The exclusion criteria were: (a) orthopedic surgery of the lower limbs in the last year (b) the use of botulinum toxin in the last 6 months; (c) presence of shortening or deformities of the ankle, knee and/or hip joints that prevented the children from keeping their feet on the ground, hindered them from maintaining the standing position, or made it impossible for them to independently perform an STS movement; and (d) difficulty maintaining an upright position without support for more than 30 seconds.

### Procedures

Inclusion and exclusion criteria were first assessed. Then, the accepted children were seated in an adjustable (i.e. height and inclination) chair without back support; hips, knees, and ankles were at 90°, and the feet were on a *BERTC System 400* (*EMG System* do Brasil^(r))^ force platform, with an acquisition frequency of 100 Hz. Once seated, they were instructed to assume an upright position, without support of the upper limbs and at a self-selected speed. At the beginning of the activity, the feet were aligned with the hips, and the hands were placed on the thighs. A circle was drawn in the center of the platform, wherein the feet were placed before each attempt, which ensured the consistency of the initial positioning of the children's feet. After this initial positioning, the children were free to make the necessary adjustments to perform the task.

After STS movement assessment, the children were assessed in an upright posture to assess the correlation between static and dynamic postural control. They stood on the platform for 30 seconds, feet aligned with the hips, upper limbs along the sides of the body and staring at a fixed point 1 meter away at the height of their eyes. Five attempts were performed: two as familiarization and warmup, followed by three valid attempts for assessment, with a 2-minute rest between each attempt.

### Data analysis

Data obtained from the force platform were processed and filtered using a digital Butterworth fourth-order low-pass filter with a 5 Hz cut-off frequency^21^ using Matlab software (*Mathworks Inc., Natick*, MA, USA). Data normalization was performed using the children's body weight values.

**Figure 1:**
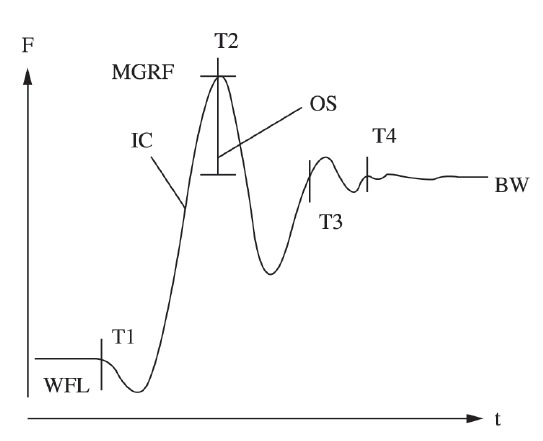
Schematic representation of the division process of the three different phases of the sit-to-stand movement. Preparation phase (T1-T2); rising phase (T2-T3); and stabilization phase (T3-T4). BW, body weight; MGRF (maximum ground reaction force); OS(overshoot); IC (incline); WFL (weight of feet/legs at rest); T1 (start of movement); T2 (seat-off); T3 (extension of body); T4 (end of movement). Source: Kralj et al.16.

The criteria for STS movement division into phases were established according to Kralj et al.^16^. For the preparation phase (F1), the beginning was determined by a decrease in vertical force greater than 2.5% relative to the weight of the feet on the platform, and the end was determined by the vertical peak force. For the rising phase (F2), measurement began with the vertical peak force on the platform and ended when the vertical force matched the body weight. The beginning of the stabilization phase (F3) was determined by the point at which the vertical force reached the body weight, and the end was determined by a vertical force oscillation of approximately 2.5% of the body weight. Details of the division of STS movement into phases are presented in Figure 1.

For each of 3 phases, the variables related to the CoP were calculated: i.e., the anteroposterior CoP displacement amplitude (Amp AP1, Amp AP2 and Amp AP3), the mediolateral CoP displacement amplitude (Amp ML1, Amp ML2 and Amp ML3), the CoP oscillation area (Area 1, Area 2 and Area 3) and the mean CoP oscillation velocity (Vel1, Vel2 e Vel3). The variables were calculated according to Duarte and Freitas^22^. The variables calculated for STS movement were also used during the standing position (i.e. Amp AP, Amp ML, Area and Vel).

Statistical analyses of the values obtained both during STS movement and while in the standing position were based on the mean value of the three attempts performed by the children for each of the variables analyzed.

### Statistical analysis

Ryan-Jones' normality test (p<0.01) was used and revealed a lack of normality for all data analyzed.

Therefore, Mann-Whitney's non-parametric test was used to compare Amp AP, Amp ML, Area and Vel variables between groups when performing STS movement. Spearman's correlation was used to assess the relationship between CoP behavior during STS movement and while in the standing position. This method was applied in each group to assess the relationships between the evaluated variables while in the standing position and during STS movement (in each of the three phases).

A significance level of 5% was adopted, and software SPSS 17 for Windows (SPSS Inc. Chicago, IL, USA) was used for the analysis.

## Results

Postural control in STS movement

**Figure 2:**
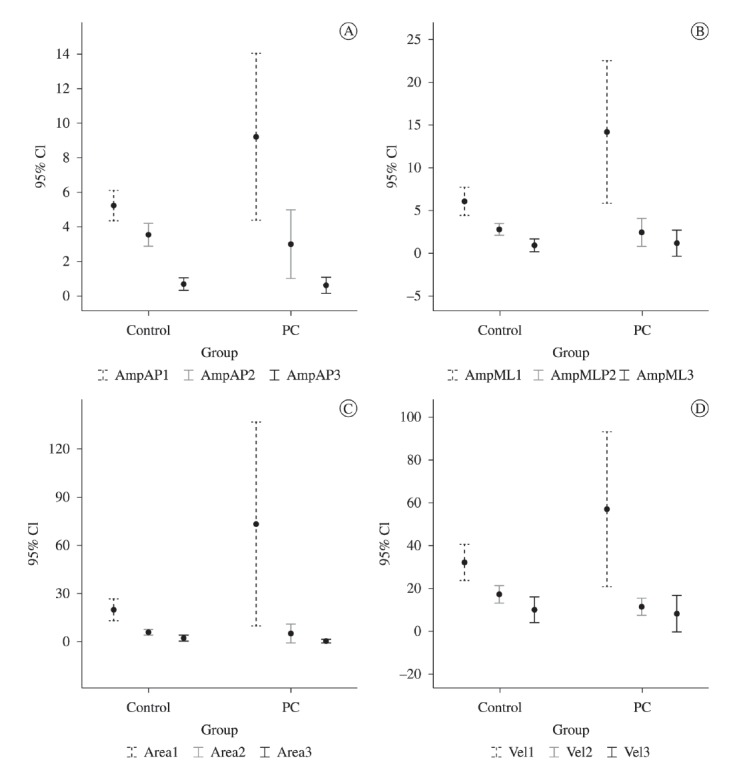
Comparison of postural control during the sit-to-stand movement between the groups of children with typical development (Control Group) and cerebral palsy (CP Group) in each of the three phases of movement. A: Antero/Posterior Amplitude of CoP displacement (AP Amp); B: Medial/Lateral Amplitude of CoP displacement (ML Amp); C: Area of CoP oscillation; D: Mean Velocity of CoP oscillation (Vel).

Significant differences in postural control were observed during STS movement between typically developing children and children with CP in the first phase (preparation phase) for the variables Amp AP (U=19.0; p=0.005), Amp ML (U=11.0; p=0.001), Area (U=12.0; p=0.001) and Vel (U=16.0; p=0.003). Children with CP exhibited higher values for these variables compared with typically developing children. The other phases of STS movement did not show significant differences between groups for the variables analyzed. A large size effect (0.92) was found, and the statistical power was 60%. The plots of these results are shown in Figure 2.

### Relationship between Static (during the Static Standing Position) and Dynamic (STS Movement) Postural Control

Typically developing children exhibited no correlations between postural control during STS movement and while in the standing position for all of the variables analyzed in all phases of STS movement (Table 1). However, children with CP exhibited moderate to strong correlations between the variables for both the standing position and STS movement in the second and third phases of STS movement (Table 2).

**Table 1:** Spearman's correlation (r) values for the sit-tp-stand movement variables in each of the three STS phases and static standing variables in the typical development group (p<0.05).

	AP Amp	ML Amp	Area	Vel
AP Amp 1	–0.12			
AP Amp 2	–0.39			
AP Amp 3	0.03			
ML Amp 1		0.21		
ML Amp 2		0.23		
Amp ML3		–0.19		
Area 1			0.17	
Area 2			–0.07	
Area 3			–0.22	
Vel 1				0.14
Vel 2				0.31
Vel 3				0.06

Antero-Posterior Amplitude of CoP displacement in phases 1, 2 and 3 of STS movement (AP Amp 1; AP Amp 2; AP Amp 3 respectively); Medio-Lateral Amplitude of CoP displacement in phases 1, 2 and 3 of STS movement (ML Amp 1; ML Amp 2; ML Amp 3 respectively); Area of CoP oscillation in phases 1, 2 and 3 of STS movement (Area 1; Area 2; Area 3 respectively); Mean Velocity of CoP oscillation in phases 1, 2 and 3 of STS movement (Vel 1, Vel 2; Vel 3 respectively); Antero-Posterior Amplitude of CoP displacement upon static standing (AP Amp); Medio-Lateral Amplitude of CoP displacement upon static standing (ML Amp); Area of CoP oscillation upon static standing (Area); Mean Velocity of CoP oscillation upon static standing (Vel).

**Table 2:** Spearman's correlation (r) values for the sit-to-stand movement variables in each of the three STS phases and static standing variables in the cerebral palsy group.

	AP Amp	ML Amp	Area	Vel
AP Amp 1	–0.15			
AP Amp 2	0.53^*^			
AP Amp 3	0.54^*^			
ML Amp 1		–0.38		
ML Amp 2		0.20		
Amp ML3		0.54^*^		
Area 1			–0.38	
Area 2			0.73^*^	
Area 3			0.60^*^	
Vel 1				–0.01
Vel 2				0.24
Vel 3				0.59^*^

* p<0.05.

## Discussion

This study revealed differences in the postural control of typically developing and CP children during the preparatory phase of STS movement. Children with CP exhibit higher CoP AP, ML, Area and Vel values in the first phase of movement^16^. During this phase, the body should make adjustments in the trunk region, initially performing flexion, with anterior displacement of the center of mass^19^, to then overcome gravity, lifting from the chair and assuming a standing position. This study also showed a correlation between static (i.e. during standing posture) and dynamic (i.e. during STS movement) postural control in the CP group.

Previous studies showed that children with CP exhibited higher values of hip flexion and anterior pelvic tilt in the beginning of STS movement when compared with typically developing children^18^^,^ 23. The strategy of flexing the trunk moves the center of mass to the support base^19^, thus reducing postural imbalance; this movement could possibly be considered an adaptive action to compensate for postural control deficits^24^. Furthermore, the head movements that follow this increased trunk flexion may stimulate vestibular receptors^25^, thus providing greater stability and control of body positioning in space. Therefore, the higher CoP oscillation in the first phase of the movement could be a compensatory adjustment used by children with CP to succeed in the task of lifting up from the chair.

In a previous study by the same research group, using a similar sample to that of the present study, Santos et al.^23^ observed that children with CP exhibited increased ankle excursion in the frontal and transverse planes during STS movement when compared with typically developing children. This increased excursion might explain the increase in AP and ML CoP displacements and determines a larger CoP oscillation area during STS movement. Studies with healthy participants show that higher values of subtalar pronation are related to increased AP and ML CoP displacements^26^. Due to the neuromotor changes children with CP display, they exhibit biomechanical misalignment of the lower limbs, with higher values of internal rotation of the tibia and femur and subtalar pronation when compared with typically developing children^23^. Therefore, children with CP, due to their biomechanical and neuromuscular limitations, increase their ankle excursion in the act of getting up^23^, possibly leading to larger AP and ML displacements, as observed in the present study. In addition, the increased ankle excursion could be interpreted as a strategy to increase the arrival of proprioceptive afferents to the central nervous system^27^, thus facilitating the maintenance of stability to perform a function.

These results may bring new prospects for rehabilitation for these children, demonstrating the importance of the ankle joint in the execution of STS movement. Based on these results, one could also make inferences regarding the need to work on functional activities during therapy to improve ankle stability and proprioception and, thus, to improve the performance of daily tasks, such as STS movement.

The CP children of this study also exhibited increased CoP oscillation velocity in the first phase of STS movement compared with typically developing children. CoP oscillation velocity is one of the main predictors of body stability, with higher values being related to greater difficulty in controlling body positioning in space^28^. Therefore, the increase in oscillation velocity observed in CP children indicates their difficulty in controlling body segments when perfoming the STS movement.

It is noteworthy that the high biomechanical demand of STS movement^14^^,^ 15 could impose restrictions on children with CP during its execution. However, even with a greater postural instability to begin the movement, the children with CP assessed in this study were able to successfully perform the movement, possibly because of their mild motor impairment.

All of the CoP differences noted between typically developing children and CP children in the first phase of STS movement indicated that the beginning of this movement could be a critical moment for children with CP. Thus, rehabilitation programs should focus on and exhaustively train the start of the getting up action to improve children's ability to anteriorly displace their center of gravity and to assume an upright position.

There were no significant differences between groups in the other phases, namely the rising phase (F2) and the stabilization phase (F3). These phases involve gravity-defying body movement (F2) and movement deceleration (F3). Therefore, they demand muscle strength and concentric-eccentric muscle control of the lower limbs, respectively, for proper interarticular coordination and for remaining in a standing position without the risk of falling. Although children with spastic hemiplegia exhibit knee-extension strength deficits^23^, which could compromise the rising phase, it was assumed that interlimb compensation occurred during the movement. In this compensation, the healthy limb acts as a support limb, providing stability and compensating the plegic limb deficits^29^^,^ 30 during the last two phases of STS movement. However, the present study did not assess postural control in specific platforms for each lower limb.

In typically developing children, unlike CP children, there was no correlation between postural control during the standing position and STS movement. A possible explanation for the lack of correlation in this group could be the less varying behavior of the postural oscillation when compared with the CP group.

There was a correlation between static and dynamic postural control in the CP group. In the CP children, the higher values of Amp AP, Amp ML and CoP oscillation Area and Vel during the standing position were related to the higher values of the same variables for STS movement (i.e., the rising and stabilization phases). This correlation was noted only in the last two phases of the movement. These phases represent when the support base, initially formed by the surface of the feet and the gluteal region, is then formed only by the individual's feet. This change may determine greater postural instability, and, thus, a more varying oscillatory behavior. In the last two phases of STS movement, the support base is similar to the base during the standing position. Therefore, one could consider the existence of common components of posture and movement of the lower limbs actively controlling these body segments, thus avoiding falls. In addition, the neuromotor deficits observed in CP^9^^,^ 31 can compromise muscle recruitment patterns^32^, resulting in postural control deficits during STS movement - especially in the final phase, which involves movement deceleration control when the individual changes from a dynamic movement to a semi-static position.

Therefore, according to the results observed, the postural oscillation of children during the standing position is related to their performance in dynamic tasks, such as STS movement. This relationship demonstrates the importance of static posture control for children with CP for performing functional tasks.

Extrapolating the conclusions of this study to rehabilitation, the authors believe the results of this study provide relevant information for clinical practice: namely, children with CP require interventions that include functional activities focused on performing the STS movement. Greater attention should be paid to the preparation phase of this movement because the main differences in postural control compared to typically developing children were observed in this phase. In addition, the correlation identified between static and dynamic postural control allows one to infer that the use of dynamic activities, such as STS, may facilitate patients' stability in the maintenance of static postures. Additionally, the training of static posture maintenance can assist in the performance of the movement of getting up.

The limitations of the study were the use of only one force platform to assess the lower limbs and the low statistical power of the tests applied. We believe that in further studies with larger samples, differences in other phases of STS movement may be identified. Furthermore, only children with mild motor dysfunction were assessed. Studies using two force platforms should be conducted to assess the relationship between postural behavior in static postures and dynamic activities in populations with greater neuromotor impairment.

## Conclusion

The children with CP assessed in this study exhibited increased CoP oscillation at the beginning of STS movement compared with typically developing children, which indicates greater difficulty of initiating the movement for children with CP. Moreover, in children with CP, increased CoP oscillation in static posture is related to increased oscillation during dynamic activities, as observed in the last two phases of STS movement. These results reveal the importance of controlling the body in the static posture to perform functional activities that put the body in motion.

## References

[1] Barela JA, Focks GMJ, Hilgeholt T, Barela AMF, Carvalho RP, Savelsbergh GJP (2011). Perception-action and adaptation in postural control of
children and adolescents with cerebral palsy. Res Dev Disabil.

[2] Verschuren O, Ada L, Maltais DB, Gorter JW, Scianni A, Ketelaar M (2011). Muscle strengthening in children and adolescents with
spastic cerebral palsy: considerations for future resistance training
protocols. Phys Ther.

[3] Tammik K, Matlep M, Ereline J, Gapeyeva H, Pääsuke M (2007). Muscle contractile properties in children with spastic
diplegia. Brain Dev.

[4] Woollacott MH, Shumway-Cook A (2005). Postural dysfunction during standing and walking in
children with cerebral palsy: what are the underlying problems and what new
therapies might improve balance?. Neural Plast.

[5] Carlberg EB, Hadders-Algra M (2005). Postural dysfunction in children with cerebral palsy:
some implications for therapeutic guidance. Neural Plast.

[6] Ostensjø S, Carlberg EB, Vøllestad NK (2004). Motor impairments in young children with cerebral palsy:
relationship to gross motor function and everyday activities. Dev Med Child Neurol.

[7] Ju YH, You JY, Cherng RJ (2010). Effect of task constraint on reaching performance in
children with spastic diplegic cerebral palsy. Res Dev Disabil.

[8] Liao HF, Hwang AW (2003). Relations of balance function and gross motor ability
for children with cerebral palsy. Percept Mot Skills.

[9] Burtner PA, Woollacott MH, Qualls C (1999). Stance balance control with orthoses in a group of
children with spastic cerebral palsy. Dev Med Child Neurol.

[10] Donker SF, Ledebt A, Roerdink M, Savelsbergh GJP, Beek PJ (2008). Children with cerebral palsy exhibit greater and more
regular postural sway than typically developing children. Exp Brain Res.

[11] Ferdjallah M, Harris GF, Smith P, Wertsch JJ (2002). Analysis of postural control synergies during quiet
standing in healthy children and children with cerebral palsy. Clin Biomech (Bristol, Avon).

[12] Rose J, Wolff DR, Jones VK, Bloch DA, Oehlert JW, Gamble JG (2002). Postural balance in children with cerebral
palsy. Dev Med Child Neurol.

[13] Pavão SL, Santos AN, Woollacott MH, Rocha NACF (2013). Assessment of postural control in children with cerebral
palsy: a review. Res Dev Disabil.

[14] Seven YB, Akalan NE, Yucesoy CA (2008). Effects of back loading on the biomechanics of
sit-to-stand motion in healthy children. Hum Mov Sci.

[15] Yoshioka S, Nagano A, Hay DC, Fukashiro S (2009). Biomechanical analysis of the relation between movement
time and joint moment development during a sit-to-stand task. Biomed Eng Online.

[16] Kralj A, Jaeger RJ, Munih M (1990). Analysis of standing up and sitting down in humans:
definitions and normative data presentation. J Biomech.

[17] Riddiford-Harland DL, Steele JR, Baur LA (2006). Upper and lower limb functionality: are these
compromised in obese children?. Int J Pediatr Obes.

[18] Park ES, Park CI, Lee HJ, Kim DY, Lee DS, Cho SR (2003). The characteristics of sit-to-stand transfer in young
children with spastic cerebral palsy based on kinematic and kinetic
data. Gait Posture.

[19] Yonetsu R, Nitta O, Surya J (2009). "Patternizing" standards of sit-to-stand movements with
support in cerebral palsy. NeuroRehabilitation.

[20] Santos AN, Pavão SL, Rocha NACF (2011). Sit-to-stand movement in children with cerebral palsy: a
critical review. Res Dev Disabil.

[21] Pavão SL, Santos AN, de Oliveira AB, Rocha NACF (2014). Functionality level and its relation to postural control
during sitting-to-stand movement in children with cerebral palsy. Res Dev Disabil.

[22] Duarte M, Freitas SMSF (2010). Revision of posturography based on force plate for
balance evaluation. Rev Bras Fisioter.

[23] Santos AN, Pavão SL, Santiago PR, Salvini TF, Rocha NA (2013). Sit-to-stand movement in children with hemiplegic
cerebral palsy: relationship with knee extensor torque and social
participation. Res Dev Disabil.

[24] Schultz AB, Alexander NB, Ashton-Miller JA (1992). Biomechanical analyses of rising from a
chair. J Biomech.

[25] Horak F, Shupert C, Herdman S (1994). The role of the vestibular system in postural
control. Vestibular rehabilitation.

[26] Cobb SC, Tis LL, Johnson BF, Higbie EJ (2004). The effect of forefoot varus on postural
stability. J Orthop Sports Phys Ther.

[27] Patla A, Frank J, Winter D (1990). Assessment of balance control in the elderly: major
issues. Physiother Can.

[28] Sobera M, Siedlecka B, Syczewska M (2011). Posture control development in children aged 2-7 years
old, based on the changes of repeatability of the stability
indices. Neurosci Lett.

[29] Sadeghi H, Allard P, Prince F, Labelle H (2000). Symmetry and limb dominance in able-bodied gait: a
review. Gait Posture.

[30] Gabbard C, Hart S (1996). A question of foot dominance. J Gen Psychol.

[31] Burtner PA, Qualls C, Woollacott MH (1998). Muscle activation characteristics of stance balance
control in children with spastic cerebral palsy. Gait Posture.

[32] Nashner LM, Shumway-Cook A, Marin O (1983). Stance posture control in select groups of children with
cerebral palsy: deficits in sensory organization and muscular
coordination. Exp Brain Res.

